# Metastatic Renal cell Carcinoma Presenting as a clear-cell Tumor in Tongue: A Case Report

**Published:** 2014-07

**Authors:** Hamid Abbaszadeh-Bidokhty, Mina Motallebnejad, Mahdieh Rajabi-Moghaddam

**Affiliations:** 1 *Cellular and Molecular Biology Research Center, Department of oral and maxillofacial pathology of dentistry college, Babol University of Medical Sciences, Babol, Iran.*; 2 *Dental research center, Department of oral medicine and diagnosis of dentistry college, Babol University of Medical Sciences, Babol, Iran.*; 3 *Department of Pathology of medical college, Babol University of Medical Sciences, Babol, Iran. *

**Keywords:** Metastasis, Renal cell carcinoma, Tongue

## Abstract

**Introduction:**

Metastatic lesions of the oral cavity are extremely rare, accounting for approximately 1% of all malignant oral tumors. The most common primary sources of metastatic tumors in the oral region are, from the most to the least common, the breast, lung, kidney, bone, and colon. Renal cell carcinoma accounts for nearly 3% of all adult malignancies. It usually metastasizes to the lungs, bone, adrenal glands, and regional lymph nodes. The incidence of metastasis from renal cell carcinoma to the head and neck region is very low. The tongue is considered a very rare atypical ear, nose, and throat (ENT) location for metastasis of renal cell carcinoma. The present case from Iran reports tongue metastasis of renal cell carcinoma (RCC).

**Case Report:**

The following report is based on an 80-year old male patient with a tongue lesion and ambiguous past medical history that ultimately leads to diagnosis of a metastatic RCC. We also updated a previous literature review that was published 2008. A histopathological differential diagnosis for clear-cell tumors is also discussed.

**Conclusion:**

Because of the rarity of metastatic tumors of the oral region as well as the presence of other lesions with clear cells, diagnosis of metastatic clear-cell RCC in the oral cavity can be very difficult and challenging.

## Introduction

Metastatic lesions of the oral cavity are extremely rare, accounting for approximately 1% of all malignant oral tumors. Renal cell carcinoma (RCC) is the third most common tumor that metastasizes to the head and neck region (1). Approximately 15% of RCCs metastasize to the head and neck region (2,3). The nose and paranasal sinuses are most commonly affected, followed by the oral cavity (1). The most common sites of oral soft tissues for metastases are attached gingiva followed by the tongue (4). A primary tumor of the kidney metastasizing to the tongue is very unusual (5).

The prognosis for patients with lingual metastasis of RCC is poor. Treatment of tongue metastasis is usually palliative and aims to provide patient comfort by means of pain relief and prevention of bleeding and infection. Surgical excision is recommended as the primary treatment, with emphasis on preservation of tongue structure and function. This can be followed by adjuvant radiotherapy to achieve local control of disease. Chemotherapeutic agents including fluoropyrimidines together with biological agents such as interferon-α can offer a palliative benefit in some patients with RCC (5). Newer agents such as sorafenib and sunitinib have been shown to improve progression-free survival in metastatic RCC (6,7). 

We discuss a case of RCC presenting as a clear-cell tumor in the tongue.

## Case Report

An 80-year old male presented with a swelling on the dorsal surface of the tongue which had been removed superficially once before by a dentist and recurred again after several months. Intraorally, a 1×1-cm oval-shaped, reddish, sessile, slightly indurated and painless swelling was observed on the midline of the dorsal surface of the tongue which had started bleeding over 5 months previously. The patient had hypertension, and there were no similar lesions in other parts of his body. 

A biopsy was taken from the lesion and submitted for histopathologic examination with suspicions of a reactive lesion.

***Gross examination***

An oval-shaped, creamy-to-brownish, elastic soft tissue mass, covered by epithelium, was submitted for histopatho- logic examination. The lesion measured 10×7×5 mm in size.

***Microscopic examination***

**Fig1:**
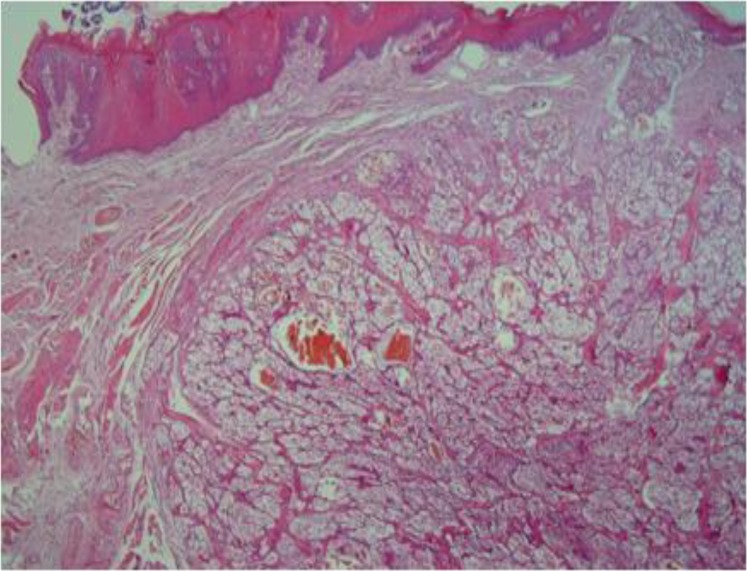
Well-circumscribed clear-cell tumor under the epithelial surface (40×)

A malignant epithelial proliferation of mostly clear cells was observed that was located as a relatively well-circumscribed nodular mass below the parakeratinized stratified squamous epithelium of the tongue mucosa (Fig. 1).

**Fig 2:**
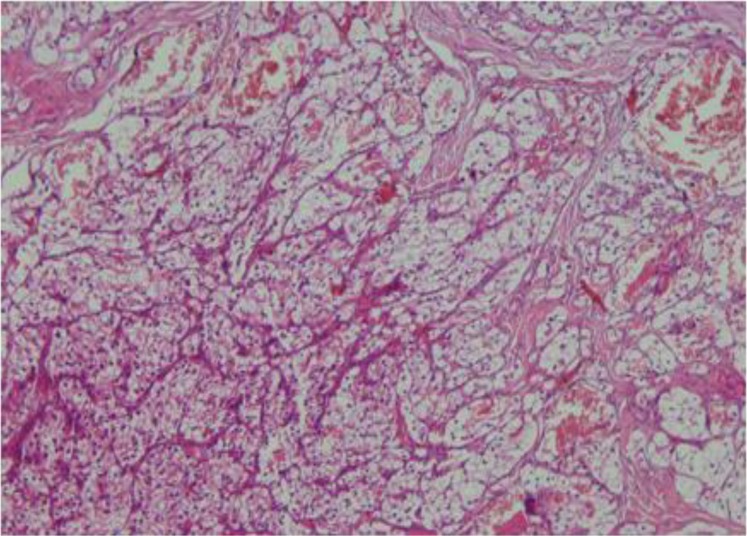
Nested pattern along with rich capillary vascular network (100×)

**Fig 3:**
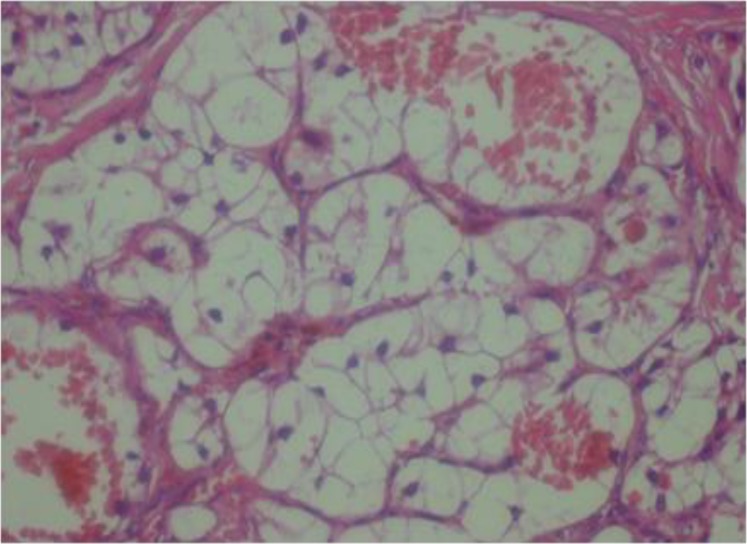
Neoplastic cells with pleomorphic nuclei and clear cytoplasm (400×)

These cells were arranged in a nested pattern and separated by fibrous septa (Fig. 2). Neoplastic cells were polygonal, had pleomorphic vesicular to hyperchromatic nuclei, were located mostly eccentrically, and had clear and sometimes eosinophilic cytoplasm with sharply outline cell membranes (Fig. 3). There was a rich capillary vascular network at the surface.

***Diagnosis***

According to histopathologic examination, primary and metastatic clear-cell tumors were suspected. Systemic examination of the patient revealed that there had not been any similar lesion in other parts of his body. The patient-stated past medical history had no notable features. Because of this, the possibility of a metastatic clear-cell tumor was dismissed.

**Fig 4:**
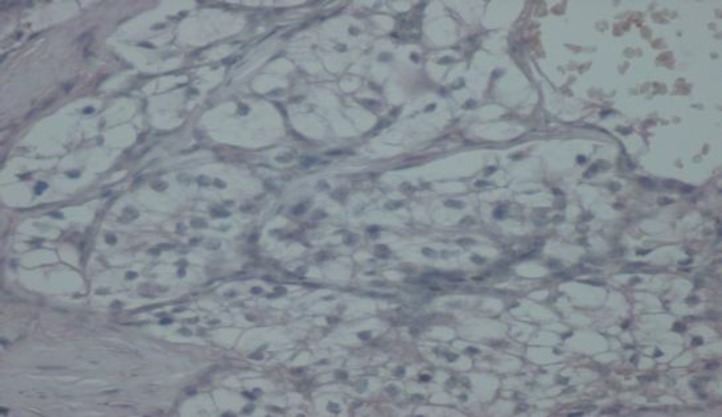
Mucicarmine staining

**Fig 5:**
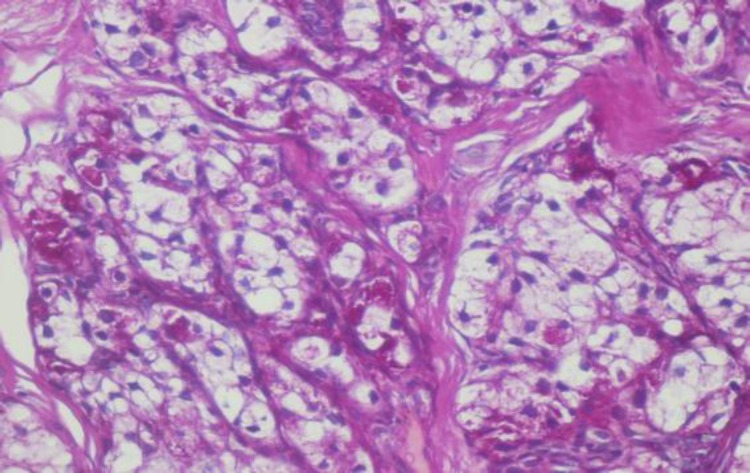
PAS staining

**Fig 6:**
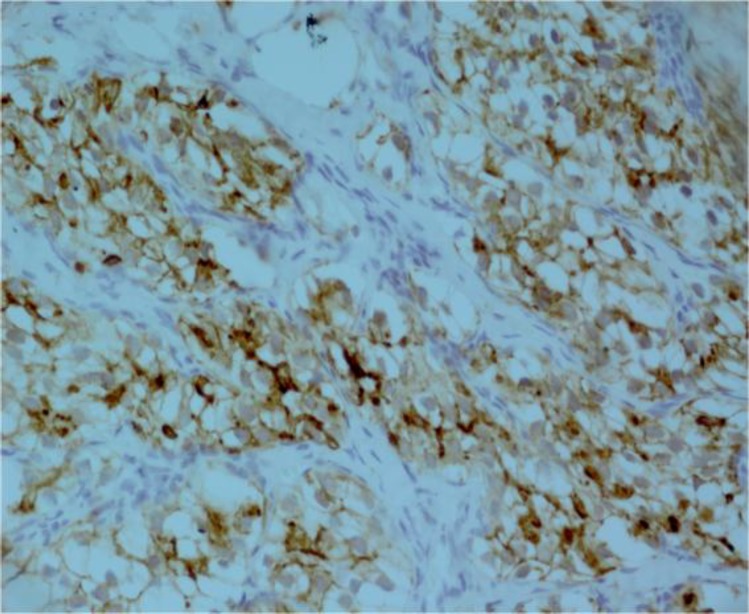
CD10 staining

The tumor was initially thought to be a primary tongue cancer with salivary gland neoplasms, especially mucoepidermoid carcinoma and acinic cell carcinoma. Mucicarmine and periodic acid Schiff (PAS) staining were performed to assess the presence of mucous cells and PAS-positive glycogen-containing cells. Mucicarmine staining became negative and PAS staining was highly focally positive (Figures 4 and 5). Therefore, these staining patterns did not prove conclusively an origin in the salivary gland. In searching for a diagnosis, we performed immunohistochemical staining for cytokeratin 7 (CK7). CK7 staining became negative, ruling out salivary gland origin. We began to suspect a metastatic clear-cell tumor such as RCC, and further investigated the patient’s history. The patient was invited to the oral and maxillofacial department once again. On this occasion, the patient mentioned a history of nephrectomy after we asked him about his renal status. Inspection of his previous pathology report showed that the patient had had a radical nephrectomy 4 years previously and the diagnosis was RCC (clear-cell variant). To confirm a diagnosis of RCC which had metastasized to the tongue, we ordered immunohisto- chemical staining for CD 10 marker; CD10 is a good marker for RCC to distinguish it from other clear-cell-type carcinomas. Immunohistochemically, tumor cells were positive for CD10 (Fig.6). 

To complete the immunohistochemistry panel, Melan-A staining was performed in order to rule out clear-cell melanoma. Melan-A staining also became negative. Therefore, we made a diagnosis of clear-cell tumor suggestive of “metastatic renal cell carcinoma”. 

***Treatment and follow-up***

A CT scan of the neck, chest, and abdomen revealed no other metastatic disease. Therefore, the patient underwent surgical re-excision of his lesion in order to provide comfort and prevent bleeding and infection. Afterwards, the patient was referred to an oncologist for systemic therapy. Subsequently, the patient was treated with sunitinib and sorafenib for his metastatic disease. The patient was still alive with no recurrence of his metastatic tongue lesion after 6 months of treatment.

## Discussion

To the best our knowledge, this case report is the first in Iran to report RCC with tongue metastasis. After a literature search using Google Scholar, we found only three cases with tongue metastasis of RCC since 2008. Before 2008, Azam et al. (5) reported 29 cases since 1911 in their literature review. Thus, the present case is the 33rd case globally reporting RCC with tongue metastasis. We completed the literature review of Azam et al (Table. 1). 

Clinically, metastatic lesions in the oral cavity may resemble reactive lesions such as pyogenic granuloma. Yoshitomi et al. (8) reported a lingual metastasis of RCC that they mistook for a pyogenic granuloma both clinically and histopathologically. Clinically, we also made a differential diagnosis of pyogenic granuloma. 

The previous history of RCC is very helpful in making a diagnosis of a metastatic lesion to the oral cavity with a renal origin. Sometimes lingual metastasis is an initial presentation of RCC, as in case reports by Azam et al. (5) and Yoshitomi et al. (8), which make the diagnosis more complicated. In our case, incompleteness of the past medical history and unawareness of past radical nephrectomy and RCC clouded our diagnosis. 

In this case, a clear-cell tumor was involved. Clear-cell tumors of the oral cavity include clear-cell variants of salivary gland neoplasms, clear-cell variants of odontogenic tumors (e.g. Pindborg tumor), primary and metastatic melanomas, and metastatic clear-cell carcinomas (e.g. RCC). Clear-cell variants of salivary gland neoplasms involve a broad spectrum, including mucoepidermoid carcinoma, acinic cell carcinoma, myoepithelial carcinoma, epithelial-myoepithelial carcinoma (double clear-cell variants) and clear-cell carcinoma. Histopathologic features such as mild pleomorphism and well-circumscription are more compatible with a low-grade mucoepidermoid carcinoma, acinic cell carcinoma and, to some extent, with clear-cell carcinoma, but not myoepithelial carcinoma and epithelial-myoepithelial carcinoma. The presence of cells similar to the mucous cells and even epithelioid cells in this case was suggestive of a low-grade mucoepidermoid carcinoma. Eccentrically located nuclei suggested a acinic cell carcinoma but not a clear-cell carcinoma. With respect to location, a Pindborg tumor was eliminated, while, according to a histopathologic view, clear-cell melanoma was a remote diagnosis. Therefore, the most likely differential diagnoses included: 1) clear-cell variant of salivary gland neoplasms, especially mucoepidermoid carcinoma and acinic cell carcinoma; and 2) metastatic clear-cell variant of RCC. As stated before, we ultimately made a diagnosis of a metastatic RCC with respect to previous history of RCC, histopathologic features, and the immunohistochemical profile. 

Because of different management approaches required for primary and metastatic cancers of the tongue, a comprehensive assessment to distinguish between them is essential. 

Generally, there is agreement that the management of tongue metastasis of RCC is palliative therapy, and therefore surgical excision with preservation of tongue function is the primary treatment. This treatment is performed to provide pain relief and prevent bleeding and infection. Following Azam et al. (5) and Yoshitomi et al. (8) and other reports, we also excised the tongue lesion to provide patient comfort.

With regard to other treatment modalities, there is some controversy. Although RCC is not radiosensitive, some advocate radiation therapy to achieve a better local control of microscopic disease. Azam et al. described postoperative radiotherapy to the oral cavity, while, like us, Yoshitomi et al. (8), did not consider radiotherapy.

Over the past 7 years, systemic therapy for metastatic RCC has changed considerably. Since metastatic RCCs do not respond to chemotherapy, cytokine immunotherapy such as interferon-α (IFN-α) has been used. Recently, targeted therapy directed against vascular endothelial growth factor (VEGF) and its elements has been introduced and provided more benefit than historical immunotherapy. Azam et al. (5) treated their patient with interferon-alpha. Yoshitomi et al. (8) followed surgical treatment of their patient by interferon-α, but due to increase of metastatic foci, they treated the patient again with targeted therapy using sunitinib and sorafenib (anti-VEGF agents). After surgical management, we treated the patient with sunitinib and sorafenib. 

Basely et al. (9) suggested that F-18 fluorodeoxyglucose positron emission tomography (F-18 FDG PET), as an imaging modality, can be a useful tool in the follow-up of RCC.

## Conclusion

Tongue metastasis from RCC is a rare condition. Differentiation among clear-cell tumors can be challenging, and it can be especially difficult to distinguish between metastatic RCC and clear-cell tumors of the salivary glands. When encountering with a tongue lesion, a thorough evaluation should be made to distinguish between primary and metastatic cancers, as the management and prognosis vary.
